# Responses of Soil C, N, P and Enzyme Activities to Biological Soil Crusts in China: A Meta-Analysis

**DOI:** 10.3390/plants13111525

**Published:** 2024-05-31

**Authors:** Zhi Yang, Yong Yuan, Jinjin Guo, Jinxi Li, Jianhua Li, Hu Yu, Wen Zeng, Yinhong Huang, Liyun Yin, Fulian Li

**Affiliations:** 1 Faculty of Modern Agricultural Engineering, Kunming University of Science and Technology, Kunming 650500, China; yz15752794251@163.com (Z.Y.); ljx3140354393@outlook.com (J.L.); 15287555035@163.com (J.L.); yuhu218323@163.com (H.Y.); 18087177486@163.com (W.Z.); hyh5468@foxmail.com (Y.H.); yinliyun2022@126.com (L.Y.); 15812232904@163.com (F.L.); 2 Yunnan Provincial Field Scientific Observation and Research Station on Water-Soil-Crop System in Seasonal Arid Region, Kunming University of Science and Technology, Kunming 650500, China

**Keywords:** BSCs, soil nutrients, enzyme activity, meta-analysis, influence factor

## Abstract

Biological soil crusts (BSCs) are often referred to as the “living skin” of arid regions worldwide. Yet, the combined impact of BSCs on soil carbon (C), nitrogen (N), phosphorus (P), and enzyme activities remains not fully understood. This study identified, screened and reviewed 71 out of 2856 literature sources to assess the responses of soil C, N, P and enzyme activity to BSCs through a meta-analysis. The results indicated that BSC presence significantly increased soil C, N, P and soil enzyme activity, and this increasing effect was significantly influenced by the types of BSCs. Results from the overall effect showed that soil organic carbon (SOC), total nitrogen (TN), available nitrogen (AN), total phosphorus (TP), and available phosphorus (AP) increased by 107.88%, 84.52%, 45.43%, 27.46%, and 54.71%, respectively, and four soil enzyme activities (Alkaline Phosphatase, Cellulase, Sucrase, and Urease) increased by 93.65–229.27%. The highest increases in SOC, TN and AN content occurred in the soil covered with lichen crusts and moss crusts, and significant increases in Alkaline Phosphatase and Cellulase were observed in the soil covered with moss crusts and mixed crusts, suggesting that moss crusts can synergistically enhance soil C and N pool and enzyme activity. Additionally, variations in soil C, N, P content, and enzyme activity were observed under different environmental settings, with more pronounced improvements seen in coarse and medium-textured soils compared to fine-textured soils, particularly at a depth of 5 cm from the soil surface. BSCs in desert ecosystems showed more significant increases in SOC, TN, AN, and Alkaline Phosphatase compared to forest and grassland ecosystems. Specifically, BSCs at low altitude (≤500 m) with an annual average rainfall of 0–400 mm and an annual average temperature ≤ 10 °C were the most conducive to improving soil C, N, and P levels. Our results highlight the role of BSCs and their type in increasing soil C, N, P and enzyme activities, with these effects significantly impacted by soil texture, ecosystem type, and climatic conditions. The implications of these findings are crucial for soil enhancement, ecosystem revitalization, windbreak, and sand stabilization efforts in the drylands of China.

## 1. Introduction

Biological soil crusts (BSCs, or biocrusts) that exist within a few centimeters of the top of the soil surface are composed of algae, lichens, mosses, microorganisms, and microarthropods [1]. About 40% of the global terrestrial ecosystem is dry land, and these areas have strong climate variability, large temperature fluctuations, and scarce precipitation [2]. In the arid areas of northwest China with harsh natural conditions and severe desertification, BSCs show unique and tenacious life characteristics and play an important ecological role in desert ecosystems [3]. In some arid regions, the coverage area of BSCs is more than 70%, which is regarded as an important part of dryland coverage [4]. In arid areas, soil texture and surface morphology are often determined by BSCs, which have the effect of affecting the hydrological cycle [5] and intercepting resources (such as soil, organic matter, seeds, and nutrient-rich dust) [6,7]. BSCs further improve soil fertility by immobilizing C [8,9], N [10], and P [7] and secreting them into the lower soil layer. In addition, due to the increase in fertility, the substrate of enzymatic reaction increased, and the soil enzyme activity was improved [11]. These contributions are of global significance [12].

According to the succession stage and dominant components, BSCs are usually divided into four categories: algae crusts, lichen crusts, moss crusts, and mixed crusts [13]. The succession stage determines its ecological function. Algal crusts are formed in the early successional stage. They are light gray in color, usually thin, and their surface is smooth and flat. They are mainly composed of fungi, cyanobacteria, and algae [14]. Algae (early successional biocrusts) are important colonizers in unstable environments and play a pioneering role in the early stages of succession by enhancing soil stability and C, N input [15]. Compared with algal crusts, the input rates of C and N [16] and the stability of the soil surface [17] increased in the presence of lichen crusts (a form of soil crust that combines soil properties and biological properties through a long-term succession process by the interaction of biological factors of algae, lichens, mosses, and small plants with surface particles) and moss crusts (the surface is rough, the color is brown in the dry season, and it is bright green in rainy season. It has chlorophyll and can carry out photosynthesis) in the late successional stage. It can be seen that BSCs play an important role in improving soil nutrients and enhancing soil stability.

Soil enzymes, as biological indicators for evaluating the health of the soil ecosystem, mainly come from plant roots, microorganisms, and animals. They are an important soil functional medium and catalyst necessary for microbial life processes and plant growth in soil [18]. Enzymes play an important role in the C, N, and P cycle [19] by enhancing the decomposition of organic waste and promoting the formation of organic matter [20]. Soil enzyme activity may be affected by the microbial community [21], soil physical and chemical properties [22], vegetation [23], disturbance [24], succession [25], and seasonal changes [26]. In the Tengger Desert of China, recent studies have shown that BSCs could increase soil enzyme activity, but the effects vary depending on the type of crusts [27], and studies have attributed the difference in enzyme activity to changes in climatic conditions and soil properties [26]. In the past few decades, the relationship between BSCs and enzyme activity has gradually become a focus of research [18,28,29].

Although there have been extensive studies, most of them focus on field monitoring in small areas with a small number of limited samples, and studies or comparisons on regional scales are lacking. Furthermore, the comprehensive influence mechanism of BSCs on soil nutrients and enzyme activities in China is not fully understood. Hence, it is necessary to systematically understand the effects of BSCs on soil nutrients and enzyme activities. The objectives of this study were to evaluate the response of soil C, N, P and four enzyme activities to BSCs in China using a meta-analysis approach and to evaluate the source of variation by solving the following problems: (1) How do BSCs and their types affect soil C, N, P and enzyme activities compared with bare land? (2) Do soil texture and depth affect the response of soil C, N, P and enzyme activities to BSCs? (3) How do ecosystem, altitude, rainfall, and temperature affect the response of soil nutrients and enzyme activities to BSCs? The results are expected to provide decision making reference for environmental management and ecological restoration in the ecologically fragile areas of dry land in China.

## 2. Results

### 2.1. Overall Effects of BSCs on Soil C, N, P and Enzyme Activities

**Figure 1:**
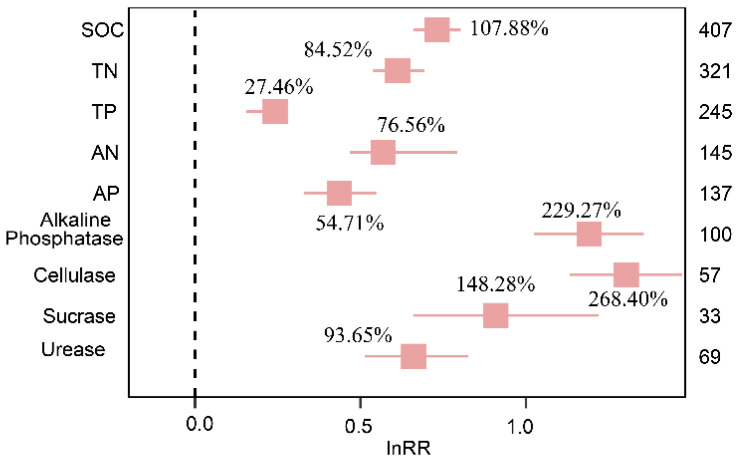
The overall effects of BSC mulching on SOC, TN, TP, AN, AP, Alkaline Phosphatase, Cellulase, Sucrase, and Urease. The label points represent the mean, and the error bars represent the 95% confidence interval. The integer value is the number of observations included in the response variable, and the percentage represents the percentage increase/decrease in the experimental group relative to the control group after conversion.

Results from the meta-analysis showed that biocrusts cover significantly (*p* < 0.05) increased C, N, P contents and enzyme activities compared to the control group without crusts (Figure 1). The contents of SOC, TN, AN, TP, and AP were increased by 107.88%, 84.52%, 45.43%, 27.46%, and 54.71%, respectively. The activities of soil Alkaline Phosphatase, Cellulase, Sucrose, and Urease were increased by 229.27%, 268.40%, 148.28%, and 93.65%, respectively. Moreover, the heterogeneity tests for soil C, N, P and enzyme activities were significant (*p* < 0.001) (Table S1), indicating that the observed values were not homogeneous, and the source of heterogeneity needs to be explained by subgroup analysis or meta-regression.

### 2.2. Effects of the Types of BSCs on Soil C, N, P and Enzyme Activities

The responses of SOC, TN, TP, AN, AP, Alkaline Phosphatase, and Cellulase to BSCs were closely related to the types of BSCs, and their between-group heterogeneity ($Q_{B}$) (Table S1) was significant (*p* < 0.05). However, soil Sucrase and Urease were not significantly affected by the type of BSC.

**Figure 2:**
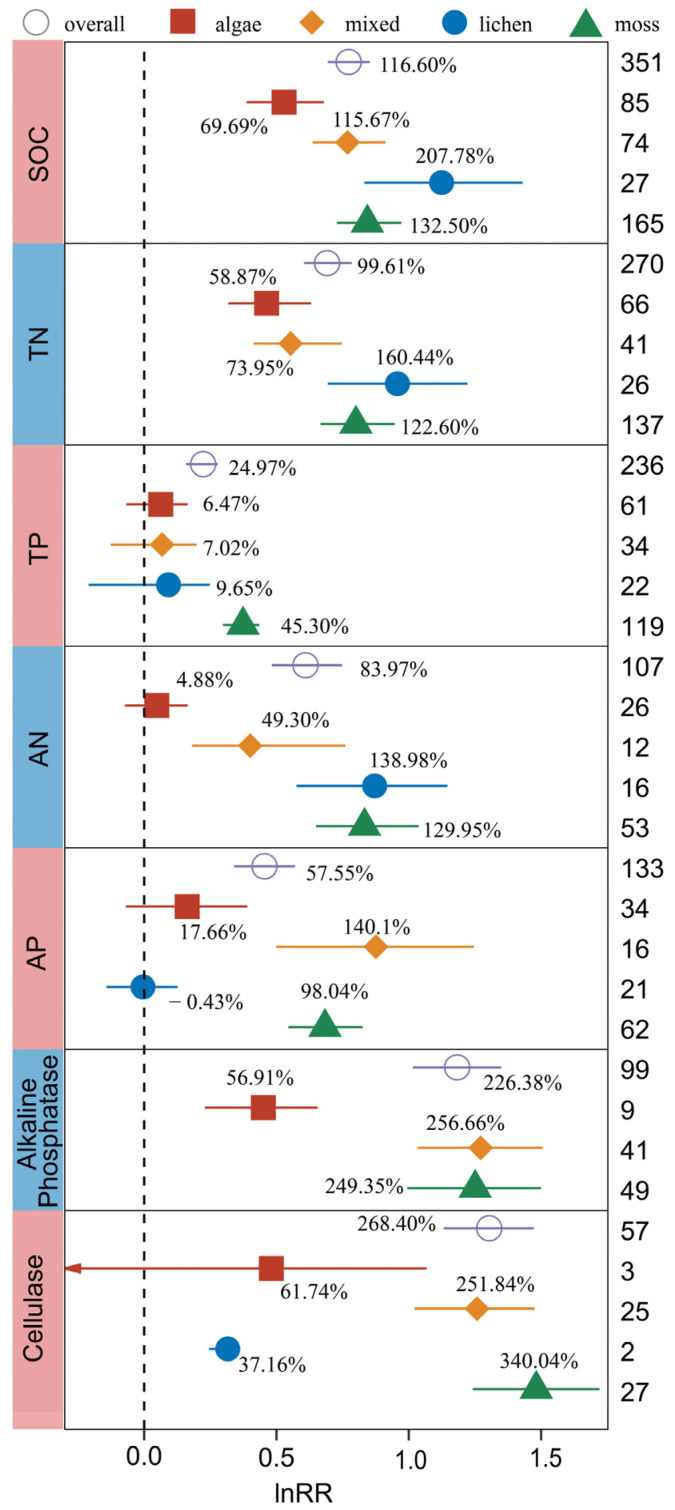
The effects of biocrust types on SOC, TN, TP, AN, AP, Alkaline Phosphatase, and Cellulase. The label points represent the mean, and the error bars represent the 95% confidence interval. The integer value represents the number of observations contained in the response variable. The percentage represents the percentage of increase/decrease in the experimental group relative to the control group after conversion. The arrow represents that the 95% confidence interval of the effect value of the study is beyond the display range of the figure.

The SOC content in the soils covered with algal crusts, mixed crusts, moss crusts, and lichen crusts increased by 69.69%, 115.67%, 132.50%, and 207.78%, respectively, indicating that the type of BSC had a significant effect on the SOC content (Figure 2). The increase under the influence of algae crusts was significantly lower than that of lichen crusts and moss crusts (*p* < 0.05). The same trend as SOC was also observed for the effect of BSC type on TN and AN content. All types of BSCs significantly affected TN content, and the influence of algal crusts differed significantly from that of lichen and moss crusts (*p* < 0.05). For AN, algal crusts had no significant effect on its content. Lichen crusts had the most significant increases in SOC, TN, and AN contents, indicating that lichen crusts could significantly improve soil C and N pools compared with other crust types. Among the four BSCs, only moss crusts could significantly increase soil TP content (45.30%). AP content increased significantly only under mixed crusts and moss crusts, with increases of 140.10% and 98.04%, respectively. Except for the level of some observations less than 10, Alkaline Phosphatase, and Cellulase in soil were significantly affected by the type of BSCs. The activities of both enzymes increased significantly under mixed crusts and moss crusts (140.10% and 98.04% for Alkaline Phosphatase, 251.84% and 340.04% for Cellulase).

### 2.3. Important Factors Affecting the Response of Soil C, N, P and Enzyme Activity to BSCs

#### 2.3.1. Soil Texture and Soil Depth

**Figure 3:**
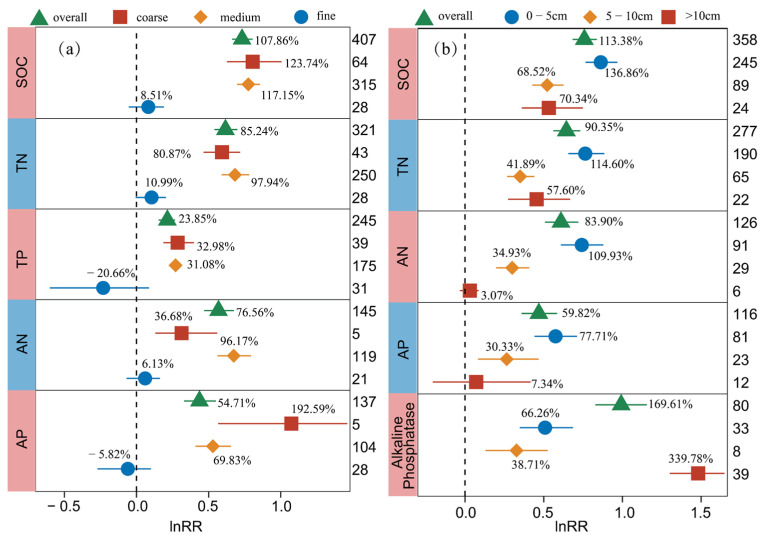
Response of SOC, TN, TP, AN, AP, and Alkaline Phosphatase to biocrust cover under the influence of soil texture (**a**) and soil depth (**b**). The label points represent the mean, and the error bars represent the 95% confidence interval. The integer value is the number of observations contained in the response variable. The percentage represents the percentage of increase/decrease in the experimental group relative to the control group after conversion.

Variation in the response of soil C, N, P and enzyme activity to BSCs under the effect of soil texture and soil depth was analyzed based on a meta-analysis approach. The results showed that the effects of BSC cover on soil C, N, P and enzymatic activity were closely related to soil texture and soil depth (Figure 3), and the $Q_{B}$ values of SOC, TN, TP, AN, and AP for soil texture and SOC, TN, AN, AP, and Alkaline Phosphatase for soil depth were significant (*p* < 0.05) (Table S1). 

The coarse- and medium-textured soil covered with BSCs significantly increased the contents of SOC, TN, TP, AN, and AP, and the fine-textured soil had no significant effect (Figure 3a). The increase in SOC in coarse soil (123.74%) was greater than that in medium soil (117.15%). Significant increases in TP and AP contents were observed in soils with a coarse and medium texture, and the increase in coarse-textured soils was generally greater than that in medium-textured soils. In contrast, the fine soil covered by BSCs resulted in a 20.66% decrease in TP and a 5.82% decrease in AP. Under the influence of fine texture, the content of TP and AP decreased, although the effect was not significant. When soil texture was used as an explanatory variable, no heterogeneity or invalid grouping of soil enzyme activity was found.

The response of soil C, N, P and Alkaline Phosphatase to BSC coverage was related to soil depth. For SOC, TN, AN, and AP, their contents in the 0–5 cm soil layer increased the most (Figure 3b). For SOC and TN, their increases in the 0–5 cm layer (136.86% for SOC, 114.60% for TN) were significantly higher than those in the 5–10 cm layer (68.52% for SOC, 41.89% for TN) and the 10–20 cm layer (70.34% for SOC, 57.60% for TN) (*p* < 0.05) (Figure 3b). On the other hand, the increases in AN and AP declined as soil depth increased. In particular, the increase in Alkaline Phosphatase in the 0–5 cm soil layer (66.26%) was significantly lower than that in the 10–20 cm soil layer (339.78%) (*p* < 0.05).

#### 2.3.2. Ecosystem Types

**Figure 4:**
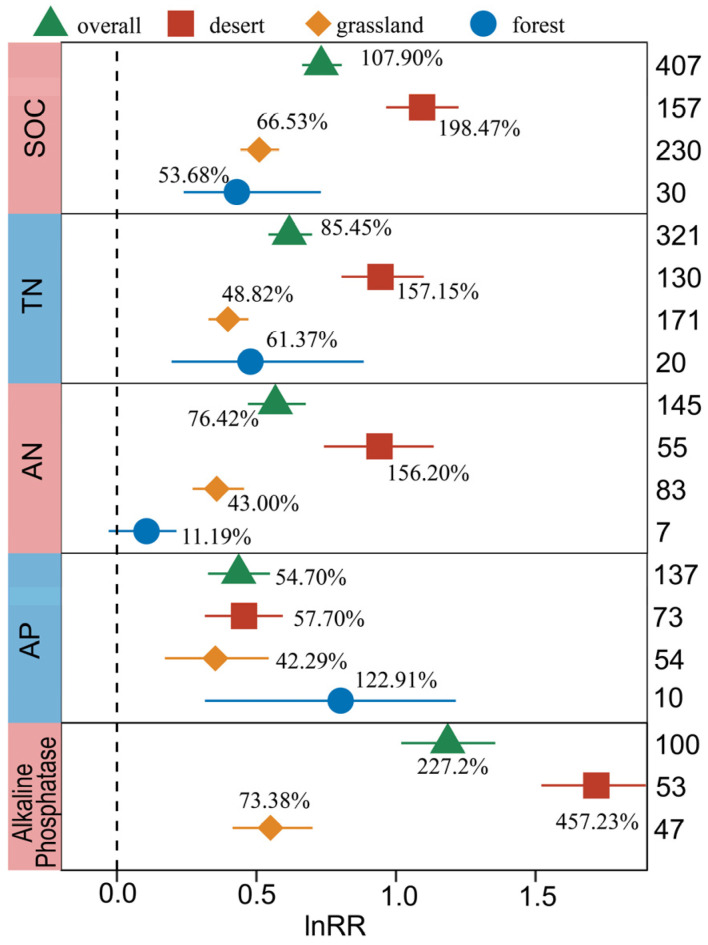
Response of SOC, TN, AN, AP, and Alkaline Phosphatase to biocrusts cover under the influence of ecosystem types. The label points represent the mean, and the error bars represent the 95% confidence interval. The integer value represents the number of observations contained in the response variable. The percentage represents the percentage of increase/decrease in the experimental group relative to the control group after conversion.

The responses of SOC, TN, AN, AP, and Alkaline Phosphatase to BSCs were closely related to the ecosystem type (Figure 4), and their $Q_{B}$ values were significant (*p* < 0.05). 

Compared with forest and grassland, the BSCs in desert ecosystems had the best effect on the increases in SOC, TN, and AN content. Specifically, the increase in SOC in desert ecosystems (198.47%) was significantly higher than that in grassland ecosystems (66.53%) and forest ecosystems (53.68%) (Figure 4). For TN, the increase order across different ecosystems was desert (157.15%) > forest (61.37%) > grassland (48.82%). A significant difference was observed in the TN increase between desert (157.15%) and grassland ecosystems (48.82%) (*p* < 0.05), similar to AN. Regarding AP, there were significant increases in all three ecosystems, with the highest observed in forest systems (122.91%), followed by desert ecosystems (57.70%) and grassland ecosystems (42.29%). The increase in Alkaline Phosphatase driven by BSCs was also significantly affected by the ecosystem type. The largest increase effect was observed in the desert ecosystem (457.23%), followed by the grassland ecosystem (73.38%), and there was a significant difference in the increase between them (*p* < 0.05).

#### 2.3.3. Altitude

**Figure 5:**
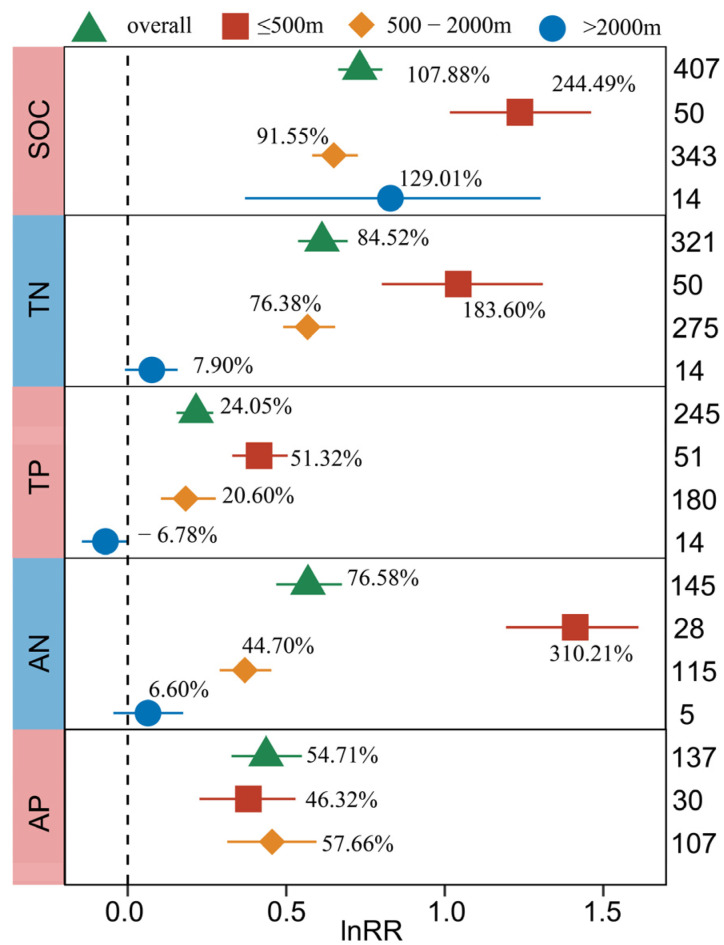
Response of SOC, TN, TP, AN, and AP to biocrusts cover under the influence of altitude. The label points represent the mean, and the error bars represent the 95% confidence interval. The integer value represents the number of observations contained in the response variable. The percentage represents the percentage of increase/decrease in the experimental group relative to the control group after conversion.

The responses of SOC, TN, TP, AN, and AP to BSCs were related to altitude, and their $Q_{B}$ values were significant (*p* < 0.05) (Table S1). Under the influence of altitude, the contents of SOC, TN, TP, and AN increased significantly at ≤500 m and 500–2000 m, and the increase rate under the two levels showed significant difference (*p* < 0.05) (Figure 5). On the whole, the above variables (SOC, TN, TP and AN) show a trend that the increase of low altitude (≤500 m) was greater than those of middle and high altitude (500–2000 m, ≥2000 m).

The component heterogeneity of SOC, TN, TP, and AN under the influence of altitude can also be explained by the continuous model, and their heterogeneity of meta-regression model values ($Q_{M}$) were significant (*p* < 0.05). Weighted meta-regression analysis showed that altitude was significantly negatively correlated with the effect values of SOC, TN, TP, and AN (*p* < 0.05) (Table S4).

#### 2.3.4. Rainfall and Temperature

As rainfall and temperature affect the formation and development of BSCs, the response of soil C, N, P and enzyme activity to BSCs under the action of annual average rainfall and annual average temperature was studied based on a meta-analysis method. The results revealed that the $Q_{B}$ values for SOC, total TN, TP, AN, AP, Alkaline Phosphatase, and Urease in relation to annual average rainfall, as well as the values for SOC, TN, TP, AN, AP, Alkaline Phosphatase, and Cellulase concerning annual average temperature, achieved statistical significance levels (*p* < 0.05) (Table S1).

**Figure 6:**
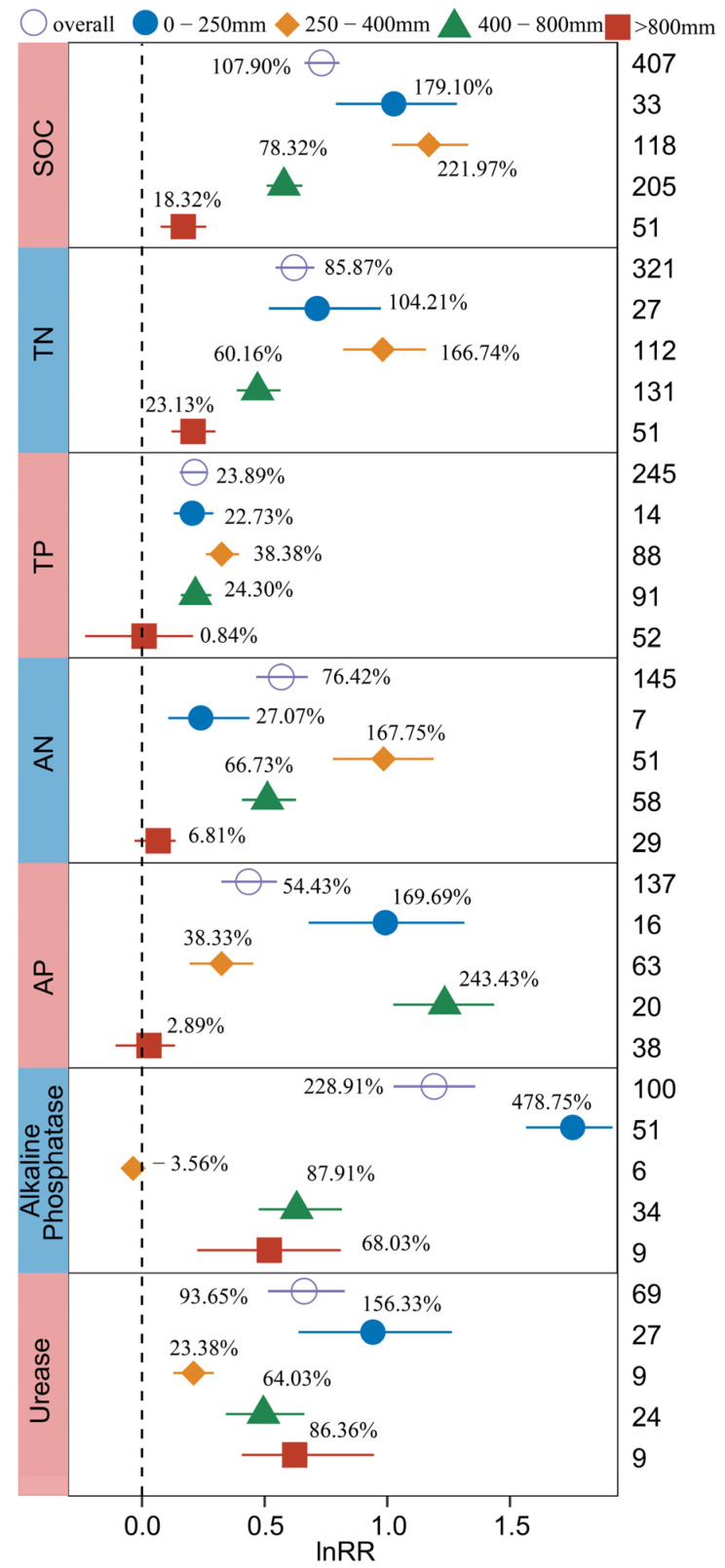
Response of SOC, TN, TP, AN, AP, Alkaline Phosphatase, and Urease to biocrusts cover under the influence of annual average rainfall. The label points represent the mean, and the error bars represent the 95% confidence interval. The integer value is the number of observations contained in the response variable. The percentage represents the percentage of increase/decrease in the experimental group relative to the control group after conversion.

The study observed a consistent trend in the increase in SOC and TN influenced by BSCs due to annual average rainfall, with the highest increases (221.97% for SOC, 166.74% for TN) occurring within the annual average rainfall range of 250–400 mm, followed by 0–250 mm, 400–800 mm, and >800 mm (Figure 6). The increase in AN in the range of 250–400 mm (167.75%) was the largest. The increase in AP in the range of 250–400 mm (38.33%) was significantly lower than those in the range of 0–250 mm (169.69%) and 400–800 mm (243.43%), respectively. The BSCs with an average annual rainfall of 0–250 mm had the greatest effect on the increase in Urease (156.33%) and Alkaline Phosphatase (478.75%).

**Figure 7:**
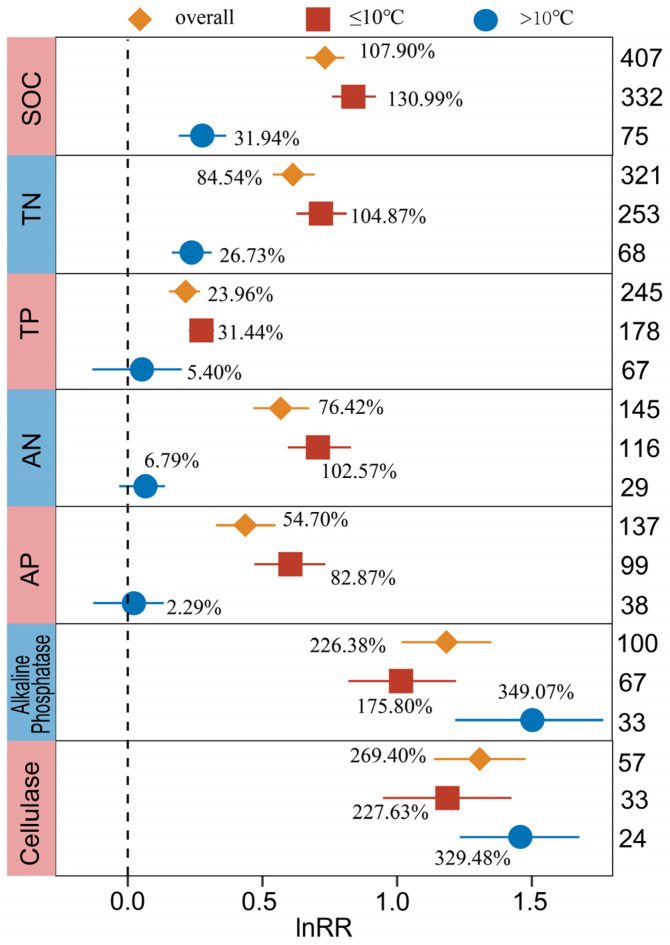
Response of SOC, TN, TP, AN, AP, Alkaline Phosphatase, and Cellulase to biocrusts cover under the influence of annual average temperature. The label points represent the mean, and the error bars represent the 95% confidence interval. The integer value is the number of observations contained in the response variable. The percentage represents the percentage of increase/decrease in the experimental group relative to the control group after conversion.

In general, the effect of BSCs on SOC, TN, TP, AN, and AP at an annual average temperature ≤10 °C was greater than that at >10 °C (Figure 7), while the effect on Alkaline Phosphatase and Cellulase was the opposite. There were significant differences in the increases in SOC and TN between an annual average temperature of ≤10 °C (130.99% for SOC, 104.87 for TN) and >10 °C (31.94% for SOC, 26.73% for TN) (*p* < 0.05). BSCs with an annual average temperature ≤10 °C significantly increased TP (31.44%), AN (102.57%), and AP (82.87%), while BSCs with an annual average temperature >10 °C had no significant effect. Soil Alkaline Phosphatase and Cellulase could be significantly increased by BSCs under different annual average temperature conditions, and the increase effect at >10 °C was significantly higher than that at ≤10 °C.

The heterogeneity of different rainfall can be explained by the continuous model, and the $Q_{M}$ values of soil SOC, TN, TP, AN, AP, Alkaline Phosphatase, and Cellulase were significant (*p* < 0.05). Weighted meta-regression analysis showed that rainfall was significantly negatively correlated with the effect values of SOC, TN, TP, AN, AP, Alkaline Phosphatase, and Cellulase (*p* < 0.05) (Table S4). This study revealed that lower rainfall led to more pronounced effects of BSCs on SOC, TN, TP, AN, AP, Alkaline Phosphatase, and Cellulase. Similarly, the heterogeneity of the annual average temperature can also be explained by the continuous model, and the $Q_{M}$ values of SOC, TN, and AN were significant (*p* < 0.05), and the effect values of these variables were also significantly negatively correlated with temperature (*p* < 0.05) (Table S4).

## 3. Discussion

### 3.1. Effects of BSCs on Soil C, N, P and Enzyme Activities

**Figure 8:**
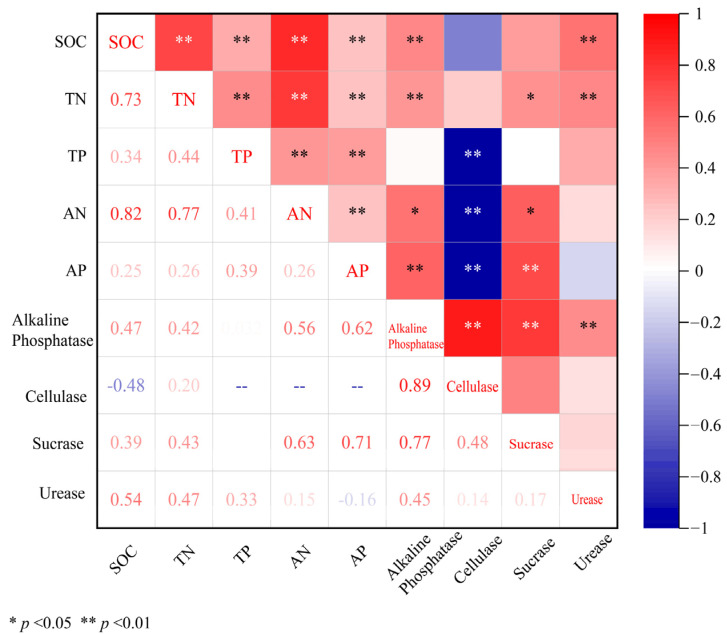
Correlation analysis among the effect sizes of soil nutrient and enzyme activity under BSCs coving.

The overall effect results showed that BSCs had a positive effect on soil nutrients (SOC, TN, AN, TP, and AP) and enzyme activities (Alkaline Phosphatase, Cellulase, Sucrose, and Urease) (Figure 1). The increase in soil nutrients and enzyme activity driven by BSCs is consistent with the results of some studies [30,31]. BSCs have a strong ability to proactively and passively fix nutrients [32]. In the initiative case, BSCs enhanced the chemical weathering of the underlying soil, fixed nutrients, and participated in the C and N cycle of the planted surface soil [33]. Since BSCs are in close contact with the soil mineral interface during growth and they secrete exopolymers, metabolites, and enzymes, they change the soil biochemical environment [18]. In the case of passive fixation, through the accumulation of dust, fine particles, including dust-bound nutrients and even organic matter, are deposited on the surface in the form of dry deposition or wet deposition, and remain in the soil under the hydration of BSCs [34]. Through Pearson correlation analysis of the effect values of soil nutrient indicators and enzyme activity indicators, we found that there were strong correlations between the indicators, and most of them were positive. This shows that under the presence of biological crusts, the increase in the content of one indicator may also promote an increase in the content of other indicators (Figure 8). Therefore, under the initiative and passive effects of BSCs, the contents of C, N, P and enzyme activities in soil were improved.

SOC content increased significantly in the environment covered by BSCs (Figure 1), although the effects varied depending on the crust type and other external conditions. On the one hand, the coverage of BSCs changes the surface morphology. When the soil surface is rough, the retention time of water in the soil is greatly increased, thus promoting water infiltration. When the soil is moist, the ability to clog soil pores or cover the soil surface will greatly reduce the infiltration rate, thus affecting evaporation loss [35,36]. Biocrusts increase the storage potential of C [37] by improving the soil hydrological cycle and helping soil to construct other organisms (such as mosses, lichens, cyanobacteria, microfungi and plants), thus creating more favorable conditions for photosynthesis. On the other hand, BSCs themselves secrete extracellular polysaccharides (EPSs) to form a filament network to help soil create aggregates to construct the macrostructure of the soil, increase the stability of the soil to erosion and other degradation factors [38], and promote the degradation of organic waste. These interactions lead to rapid nutrient accumulation, improved soil C cycling, and increased SOC content.

Soil TN and AN were also significantly increased under BSCs (Figure 1). In the process of BSCs nitrogen fixation, algae play a major role. The influence of the environment on the nitrogen fixation process is carried out in layers. Moisture is a prerequisite for nitrogen fixation and a primary control of nitrogen fixation. Because BSCs improve the hydrological conditions, which provides favorable conditions for the nitrogen fixation of microorganisms in soil. The light intensity is an important driving factor for nitrogen fixation (diazotization) by algae crusts [10]. Under sufficient hydration, the photochemical process is the source of its nitrogen fixation energy [10]. In addition, studies have shown that BSCs are emitters of nitric oxide (NO) and nitrite (HONO), and BSCs dominated by dark blue algae obtain the largest flux, which is 20 times that of adjacent non-crusts soil [39]. It is precisely because of the existence of BSCs that the N cycle is closely coupled with microbial activities, which increases the efficiency of microbial nitrogen fixation and increases the soil N content. Because AN can be absorbed and utilized by plants in the near future, it may lead to a lower degree of increase than TN. The crusts increase the N content in the soil by improving the biochemical environment of microorganisms.

Soil P was also increased under BSCs (Figure 1). Studies have shown that with the development of BSCs, soil microbial abundance can be improved [40]. Soil microorganisms are the determinant of P speciation and the key driver of P transformation. The Alkaline Phosphatase involved in the hydrolysis of orthophosphate is usually derived from soil microorganisms [41,42]. It is precisely because of the increase in the number and diversity of microorganisms that Alkaline Phosphatase activity increased, OP (NaHCO_3_-Po) mineralization accelerated, and AP content increased. Additionally, the deposition of dust in the atmosphere will also increase AP. With the succession of crusts, its roots gradually develop, which can promote the release of NaHCO3-Po [43]. Under the combined action of the increase in soil microbial abundance and the accumulation of dust in the atmosphere, soil phosphorus accumulated.

Similarly, the succession of BSCs also increased the activity of enzymes in soil (Figure 1). The soil enzyme is secreted into the soil by microorganisms. Enzymes in soil are also a kind of catalyst for microorganisms to obtain energy and nutrients. With the development of BSCs and the accumulation of soil nutrients, the substrate of enzymatic reaction increased, thus increasing the enzyme activity [44]. Studies have shown that BSCs can absorb solar radiation through their dark pigments to slow down climate fluctuations, thereby increasing enzyme activity [45]. The higher the BSC development level or biomass and coverage, the higher the soil enzyme activity. In addition, the differences in crusts’ community composition and development degree cause changes in soil physical, chemical, and biological properties, which will affect soil microorganisms and enzyme activities to a certain extent [46]. With the succession of BSCs, soil properties and microbial communities also changed synergistically, and soil enzyme activities increased.

The subgroup analysis of BSC types showed that the increases in C, N, P and enzyme activity in lichen and moss crusts were greater than those in algae crusts (Figure 2). The succession of BSCs determines its ecological function, which plays an important ecosystem function in development, coordination of decomposition and mineralization, regulation of nutrient availability, and primary productivity [47]. In the early succession of BSCs, due to poor soil stability, low species abundance, and low soil nutrient input rate, there was only a single crust type and low coverage. With the progress of succession, the input rate of C and N and the stability of soil surface increased, and the accumulation of soil nutrients accelerated in the presence of lichens and mosses in later succession [16]. In addition, the succession of BSCs can change the microbial community. Studies have shown that microbial richness reaches the highest level in mosses at the late stage of succession [48]. The study in the Northern Cape of South Africa showed that the succession of BSCs increased the microbial abundance and diversity of dryland ecosystems and further affected nutrient cycling [49], which was consistent with the results of our meta-analysis. 

Further research suggests that the early successional algae crusts are lighter in color, have almost no anti-ultraviolet pigment deposition, and have a lower biomass. As the succession progresses, lichens and moss crusts appear, and some pigments (such as canthaxanthin, β-carotene, and pseudocladin) contained in them are important for ultraviolet energy protection and prevent the destruction of photosynthetic pigment II (PSII) and protein complexes [50]. The dark color will also increase the soil temperature, so that the soil C, N, P transformation and plant absorption occur faster. In addition, the larger biomass generated in the later succession can also better stabilize the soil and prevent wind erosion and water erosion, thereby maintaining soil fertility [51]. 

Based on the above analysis, we can conclude that with the succession of BSCs, lichen crusts and moss crusts become the dominant crust types, their higher biomass weakens soil erosion and increases soil stability and soil C, N, P content. The improved soil conditions were conducive to the succession and reproduction of organisms in the surface soil, thus improving soil enzyme activity. With the mutual promotion of the BSCs and the soil, the stability of the ecosystem can be improved. This finding has great value for studying environmental evolution and ecological restoration.

### 3.2. Important Factors Affecting the Response of Soil C, N, P and Enzyme Activity to BSCs

Based on the results of subgroup analysis, the soil characteristics (texture and soil depth), ecosystem type, and climatic conditions (altitude, rainfall and temperature) were found to significantly affect the response of soil C, N, P and enzyme activity to BSCs. To better understand how BSCs drive changes in soil nutrients and enzyme activities, the influences from these factors are discussed as follows.

#### 3.2.1. The Response of Soil C, N, P and Enzyme Activity to BSCs under the Effect of Soil Texture and Soil Depth

According to the subgroup analysis of soil texture, we found that the contents of C, N, and P in coarse soil and medium soil increased significantly under the coverage of BSCs, while the fine soil increased less or even not significantly (Figure 3a). Soil texture determines the soil tillage and water retention, fertilizer retention, ventilation and water permeability. Due to the different soil texture, the soil fertility traits, fertilizer supply status, and root growth environment are different. The influence of soil texture on plants mainly lies in its influence on the spatial and temporal changes and morphological distribution of plant roots, which in turn affects the root activity, root matter accumulation, and accumulation rate [52]. The coarse- and medium-textured soil have a good structure and strong water and fertilizer retention capacity, which is conducive to the development of hyphae and rhizoids. The fine-textured soil has strong viscosity, great plasticity, and a compact structure, which is not conducive to the development of mycelium and rhizoid. This is also the reason why BSCs developed under coarse and medium soil conditions have better C, N, and P increase effects than BSCs developed under fine soil conditions. Another reason may be that due to different geographical locations, fine soil is mostly distributed in areas with abundant precipitation. Compared with dry areas, the nutrient content of bare land in wet areas is higher, and the nutrient accumulation is not obvious even the presence of BSCs. In addition, studies have shown that the finer soil with higher water holding capacity promotes the growth of BSCs under colder climatic conditions, while the negative impact was found under hot climatic conditions [53,54]. The high temperature and high humidity environment will reduce the photosynthetic capacity of plants, thus inhibiting the distribution of crusts. Therefore, soil texture directly affects soil permeability and hydrothermal conditions, and indirectly affects the succession of BSCs, thereby changing soil C, N, P content and enzyme activity.

The results of subgroup analysis showed that the effect of BSCs on the increase in soil C, N and P decreased gradually with the deepening of soil depth, and the highest increase effect was observed in the surface layer of 0–5 cm (Figure 3b). The reason for this phenomenon may be related to soil water content. After rainfall, the available water in the surface soil is sufficient to activate carbon sequestration components and drive photosynthesis to produce ATP and carbohydrates for nitrogen fixation [55]. The contribution of BSCs to soil nutrients is limited to shallow depths [56]. Due to the existence of BSCs in shallow soil, soil evaporation is low, and the infiltration time after rainfall is long, which is also conducive to soil water retention [57]. The superior light and heat conditions in the shallow layer are also more conducive to the photosynthesis of the crusts and the accumulation of shallow soil nutrients. Furthermore, the accumulation of materials (such as dust) in the soil surface also increased the distribution of nutrients in the soil profile. The increase in Alkaline Phosphatase in the soil layer of >10 cm was the largest, which was different from the increase trend of soil P content in the profile. This may be due to the interaction of other potential effects, such as the time of BSCs succession, other plant roots, etc.

#### 3.2.2. The Response of Soil C, N, P and Enzyme Activity to BSCs under the Effect of Ecosystem Type

According to the subgroup meta-analysis of ecosystems (Figure 4), we found that the increase rate of nutrients and enzyme activities in desert ecosystems was generally higher than that in grassland ecosystems and forest ecosystems, which was consistent with other global meta-analysis results [31]. This phenomenon may be due to the increase in plant coverage in grassland ecosystems, which leads to competition between BSCs and plants for light and soil moisture, while plant shading increases the duration of wet surface conditions and affects the balance between respiration and photosynthesis of BSCs [58,59,60]. From the perspective of surface organisms, desert conditions are not conducive to the growth of other tall plants. The succession of BSCs are key to the process of desert restoration, and crusts have a significant impact on the properties of soil covered by them. In grassland and forest, the effect of BSCs on soil is weaker than that of plants. Moreover, it may be due to the different initial conditions of bare land in several ecosystems, soil from deserts is the most barren. The increased proportion of soil nutrients caused by biocrust cover in deserts is more obvious than that in the other two ecosystems.

#### 3.2.3. The Response of Soil C, N, P and Enzyme Activity to BSCs under the Effect of Climate Factors

Through the analysis of rainfall (Figure 6), we found that the increase in soil C, N, P and enzyme activity was the most significant when the annual average rainfall was 250–400 mm (semi-arid). This may be because when the rainfall is 400 mm (semi-humid, humid), surface runoff will have a negative impact on nutrient accumulation, and it is also prone to leaching. If the interaction between temperature and precipitation is considered, the development of crusts in high-temperature and high-humidity environments will be inhibited [53], resulting in blocked nutrient accumulation and decreased enzyme activity, which is consistent with our research results (Figure 5 and Figure 7). The influence of altitude is often reflected in the annual average temperature. The high temperature in the middle- and low-altitude areas provides good light and heat conditions, which is conducive to the succession of BSCs, so it is more suitable for the accumulation of C, N, P and the improvement of enzyme activity.

Based on the above analysis, we found that soil (soil texture and soil depth), ecosystem type, and climate factors were important factors affecting the response of soil C, N, P and enzyme activities to BSCs. Among them, the content of C, N, P and enzyme activity in 0–5 cm soil under medium and coarse texture were most significantly affected by BSCs. The highest increases in soil C, N, P content and enzyme activity caused by BSCs occurred in desert ecosystem. When the annual average rainfall is 250–400 mm (semi-arid) and the annual average temperature is less than 10 °C, the increases in soil C, N, P and enzyme activity were the most significant. Through the analysis of these factors, we found that these external factors mainly affect BSCs through hydrothermal conditions, thus changing soil conditions. This provides an important reference for further research on BSCs in the future.

### 3.3. Limitations and Uncertainties

Responses of soil C, N, P and enzyme activities to BSCs under the influence of various factors were systematically analyzed. However, the present study still suffers some limitations. For example, under the influence of soil depth, the variation in the increase in Alkaline Phosphatase activity (the highest value occurred in the 10–20 cm soil layer) in the profile was inconsistent with that of C, N, and P contents (the highest values occurred in the 0–5 cm soil layer), which may be due to the lack of sample size and the interaction of other effects. Additionally, the succession time of BSCs determines the thickness or density of BSCs, which is also an important factor affecting the results, but we did not explore the impact due to data limitations. A few studies have found that human activities had varying impacts on BSCs, including positive, negative, and unaffected areas [61]. Unfortunately, the number of experiments examining the impact of human activities was too limited to be included in the meta-analysis. Therefore, the mechanism of soil improvement by BSCs under various factors needs to be further investigated comprehensively.

In addition, the southern part of the Kunlun Mountains, the central region of Qinghai, and the northern part of Xinjiang in northwest China are the most favorable for the growth of BSCs in China [61]. However, perhaps due to the harsh climate and limited human development, there are few reports on BSCs in these regions, resulting in a mismatch between the distribution of BSCs and the research hotspots. It is necessary to strengthen the research on the key areas of BSC distribution. Moreover, this study did not analyze the influence of other external factors on the types of biological crusts, which should be considered in future research. Finally, the sample sites of this study are only from China, and statistical analysis on a global scale is also necessary.

## 4. Materials and Methods

### 4.1. Literature Search

To study the influence of BSCs on soil C, N, P and enzyme activities, the peer-reviewed literature published before 26 June 2023 was collected using the Web of Science (WOS, https://apps.webofknowledge.com), Chinese National Knowledge Infrastructure (CNKI, http://www.cnki.net) and Chongqing VIP Database (VIP, http://www.cqvip.com). The keywords for the literature search were: (“biological soil crusts” or “biocrusts” or “BSCs”) and “China”. The collected publications were carefully screened according to the following criteria:

(1) The effect of biocrusts on at least one interest variable was reported, i.e., SOC (if soil organic matter (SOM) was reported, SOM × 0.58 was used to convert to SOC), TN, TP, AN, AP, Alkaline Phosphatase, Cellulase, Sucrase, and Urease.
(2) A control group should be set up, and the control group should be bare land, no BSCs coverage (except for artificial removal of BSCs), quicksand, or sandy land.
(3) The mean (*Xe*), standard deviation (*SD*) or standard error (*SE*) and repetition (*n*) of the target variable can be obtained directly or can be calculated using the data provided.
(4) If the data are reported more completely in another article, the published articles and their measurement methods are excluded.
(5) Compared with the control group, there should be no other human treatment except BSC coverage (such as the addition of exogenous substances, artificial change of precipitation gradient, light intensity, etc.).

**Figure 9:**
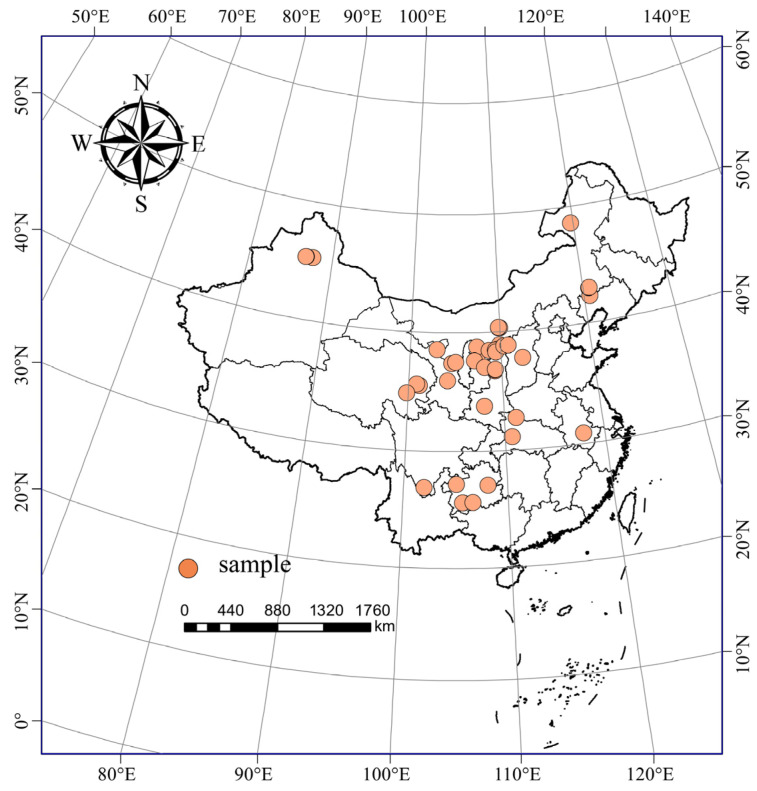
Distribution of the study sites in China included in this meta-analysis.

A total of 2856 literature studies were collected. According to the above criteria for the literature screening, the redundant and non-conforming studies were excluded. Ultimately, 71 peer-reviewed articles published between 2002 and 2022 were selected (see supplementary materials for details). The experimental studies were conducted at latitudes from 25°37′ N to 48°46′ N and longitudes from 84°31′ E to 119°45′ E. The annual mean temperature ranged from −0.3 to 20.1 °C, the annual mean rainfall ranged from 110 to 1390 mm, and the altitude ranged from 50 to 5011 m. The soil samples (experimental group and control group) in this study were from a depth range of 0–30 cm. The distribution of the study sites is shown in Figure 9.

### 4.2. Database Generation

After the publications were screened and selected, the data were collected from the text, tables, and figures of the articles. Data values presented as figures were extracted using GetData Graph Digitizer version 2.24 (http://getdata-graph-digitizer.com). The database included the mean, observed number, and standard deviation of soil C, N, P (SOC, TP, TN, AP, AN) and enzyme activities (Alkaline Phosphatase, Cellulase, Sucrase, Urease) from control (bare land with no BSCs cover, shifting sand, or sandy land) and treatment (biocrusts cover). If the number of experimental repetitions was not clearly stated, the number of sampling points was used as the number of experimental repetitions. If the standard deviation (*SD*) or standard error (*SE*) were not clearly stated, and could not be determined by other methods, it was regarded as *SE*. *SD* values were calculated from the *SE* using the following formula:

(1) $$SD=SE\cdot \sqrt{n}$$

where *n* is the number of repetitions.

In order to explain the response of soil nutrients and enzyme activities to BSCs, the following grouping variables were selected as explanatory variables:

(1) Soil texture: coarse (silt loam, loamy sand, sandy loam, sand, loam, and silt), medium (clay loam, sandy clay loam, and silty clay loam), fine (sandy clay, silty clay, and clay);
(2) Biocrusts type: algae crusts, lichen crusts, moss crusts, mixed crusts;
(3) Ecosystem type: desert ecosystem (sand and desert), grassland ecosystem (grassland, pasture, desert grassland) [31], forest ecosystem;
(4) Soil depth: depth ≤ 5 cm, 5 cm < depth ≤ 10 cm, depth > 10 cm;
(5) Annual average rainfall: rainfall ≤ 250 mm, 250 mm < rainfall ≤ 400 mm, 400 mm < rainfall ≤ 800 mm, rainfall > 800 mm [62];
(6) Annual average temperature: AT ≤ 10 °C, AT > 10 °C;
(7) Altitude: altitude ≤ 500 m, 500 m < altitude ≤ 2000 m, altitude > 2000 m.

At the same time, data sources, study sites, latitude and longitude, soil texture, climate conditions, and other information were extracted from the articles to analyze the factors influencing the responses of soil C, N, P and enzyme activities to BSCs. A total of 71 peer-reviewed papers were screened out, including 407 paired datasets of SOC, 325 paired datasets of TN, 247 paired datasets of TP, 145 paired datasets of AN, 137 paired datasets of AP, 100 paired datasets of Alkaline Phosphatase, 57 paired datasets of Cellulase, 33 paired datasets of Sucrase, and 69 paired datasets of Urease.

### 4.3. Meta Analysis

Meta-analysis was performed using MetaWin 3.0.14 software. The natural log of response ratio (*lnR*) was chosen as the effect size. For each pair of observations, the effect size and the variance *v* of each *lnR* were calculated as follows [63]:

(2) $$lnR=ln\left(X_{E}/X_{C}\right)$$

(3) $$v=\frac{SD_{E}^{2}}{n_{E}X_{E}^{2}}+\frac{SD_{C}^{2}}{n_{C}X_{C}^{2}}$$

where $X_{E}$ and $X_{C}$ were the mean values of the soil C, N, P content and enzyme activities under treatment and control, respectively; $n_{E}$ and $n_{C}$ were the number of replicates of the treatment and control, respectively; $SD_{E}$ and $SD_{C}$ were the standard deviations of the treatment and control, respectively.

Before performing a weighted meta-analysis, the frequency distribution of *lnR* (Figure S1) was plotted to test the normality of the data [64]. The weighted random effect model was used to calculate the overall effects size (*lnRR*) [65]. A bootstrapping technique with 9999 iterations was used to generate a bias-corrected 95% confidence interval (95% CI) around the effect size. If the 95% CI did not overlap with zero, the response variable was considered to respond significantly to BSCs. Compared with the control, the percentage change induced by BSCs was calculated as follows [66]:

(4) $$Change\%={(e}^{lnRR}-1)\times 100\%$$

If the percentage value calculated in the above equation was positive, it indicated that the BSC had a promoting effect on the response variable; if the percentage value was negative, it indicated inhibition.

The total heterogeneity of each variable was assessed using statistical total heterogeneity ($Q_{T}$) and $I^{2}$ index (Table S1) [67]. Subgroup analysis was performed on all data, and *Q_T_* was divided into within-group heterogeneity ($Q_{W}$) and between-group heterogeneity ($Q_{B}$). The dataset was then grouped according to the level of categorical variables with significant $Q_{B}$ (*p* < 0.05) (Table S1) [68]. If the 95% CIs did not overlap, there was a significant difference between the two levels [69].

The continuous model was used to determine the relationship between *lnR* and continuous factors such as annual average temperature, rainfall, and altitude. The parametric mixed model method was used to test whether the slope of the weighted regression deviates from zero [67]. When *p* < 0.05, the regression was considered significant.

Publication bias was tested using a variety of methods, including funnel plot (Figure S2), Kendall’s tau (Table S2), and Rosenthal’s fail-safe number (α = 0.05) (Table S3). Only when the results of the three methods were established at the same time, that is, the funnel plot clearly showed asymmetry (only for the overall data set), or the rank correlation test was significant (*p* < 0.05), and the Rosenthal fault safety number appeared less than 5*N* + 10 (*N* was the observation number), the publication bias could be confirmed [70].

## 5. Conclusions

In this study, the effects of BSCs and its associated factors on soil C, N, P and enzyme activities in China were systematically studied using a meta-analysis. The results showed that biocrust mulching significantly increased soil C, N, and P content, and greatly improved soil enzyme activities. This enhancement effect gradually increased with the succession of BSCs from algae crusts to lichen crusts and moss crusts. Overall, BSC cover increased SOC, TN, AN, TP, and AP by 107.88%, 84.52%, 45.43%, 27.46%, and 54.71%, respectively, as well as increased soil enzyme activities by 93.65–229.27%, and BSC type had significant impacts on the improvement of soil nutrients and enzyme activities. Due to the coupling effect of soil texture, ecosystem type, and climatic conditions on the succession of BSCs, there were significant differences in soil C, N, P content and enzyme activity under different environmental conditions. BSCs covered in the soil with coarse and medium texture had higher increases in SOC, TN, TP, AN, and AP content than those in the soil with fine texture, and the best increase effect occurred in the top 5 cm of soil. BSCs in the desert ecosystem had the greatest improvement in SOC, TN and AN content, compared with forest and grassland ecosystems. Moreover, the BSCs at low altitude (≤500 m) with an annual average rainfall of 0–400 mm and an annual average temperature ≤10 °C were the most beneficial for improving soil C, N, and P content. Our results suggest that BSCs play a key role in increasing soil C, N, P and enzyme activities, and soil texture, ecosystem type, and climate conditions are important factors affecting this effect. The findings have crucial implications for soil improvement, windbreak, and sand fixation, and ecosystem restoration in vulnerable areas of China, but further long-term studies are required to fully understand the mechanism of soil characteristics responding to BSCs under complex factors on a larger scale.

## Data Availability

All data supporting the findings of this study are available in the paper.

## Supplementary Materials

The following supporting information can be downloaded at: https://www.mdpi.com/article/10.3390/plants13111525/s1, Supplementary literature: A total of 71 articles were included in the Meta-analysis; Figure S1: The frequency distribution of the logarithmic response ratio of soil organic carbon (SOC), total nitrogen (TN), total phosphorus (TP), available nitrogen (AN), available phosphorus (AP) content and Alkaline Phosphatase, Cellulase, Sucrase, Urease activity to biological crusts. The curve is a fitting curve obtained by Gaussian equation fitting; Figure S2: Funnel plot of logarithmic response ratio of soil organic carbon (SOC), total nitrogen (TN), total phosphorus (TP), available nitrogen (AN), available phosphorus (AP) content and Alkaline Phosphatase, Cellulase, Sucrase, Urease activity to biological crust, funnel plot effect value (natural logarithm of response ratio, *lnR*, horizontal axis) and standard error (*SE*, vertical axis); Table S1: The overall heterogeneity (*Q_T_*) of the response of each explanatory variable to the biological crust, the percentage of heterogeneity caused by the real variation between the effect sizes (*I*^2^), and the heterogeneity between groups (*Q_B_*); Table S2: Kendall’s tau rank correlation tests for assessing publication bias; Table S3: Rosenthal’s fail-safe numbers for assessing publication bias; Table S4: The results of weighted regression analysis of the effect values (*lnR*) of response variables with annual average temperature, annual average rainfall and altitude; Table S5: Meta-analysis of initial data. References [71,72,73,74,75,76,77,78,79,80,81,82,83,84,85,86,87,88,89,90,91,92,93,94,95,96,97,98,99,100,101,102,103,104,105,106,107,108,109,110,111,112,113,114,115,116,117,118,119,120,121,122,123,124,125,126,127,128,129,130,131,132,133,134,135,136,137] are cited in the Supplementary Materials.
