# Supplementary material for: Responses of Soil C, N, P and Enzyme Activities to Biological Soil Crusts in China: A Meta-Analysis
Source: Plants (Basel). 2024 May 31;13(11):1525. doi: 10.3390/plants13111525 (PMC11174547; doi:10.3390/plants13111525)
Supplement: Supplementary file 1 [file plants-13-01525-s001.zip › Supplementary Material .pdf]

SUPPLEMENTARY INFORMATION

Responses of soil C, N, P and enzyme activities to biological soil crusts in China: a meta-analysis

Zhi Yang[1,2], Yong Yuan[1,2,*], Jinjin Guo[1,2,*], Jinxi Li[1,2], Jianhua Li[1,2], Hu Yu[1,2], Wen Zeng[1,2], Yinhong Huang[1,2], Liyun Yin[1,2], Fulian Li[1,2]

[1] Faculty of Modern Agricultural Engineering, Kunming University of Science and Technology, Kunming 650500, PR China; 2234415152@qq.com(Z.Y.); 3140354393@qq.com(J.L.); 15287555035@163.com(J.L.); yuhu218323@163.com(H.Y.); 2817303579@qq.com(W.Z.); hyh5468@foxmail.com(Y.H.); yinliyun2022@126.com(L.Y.); 2844755739@qq.com(F.L.)

[2] Yunnan Provincial Field Scientific Observation and Research Station on Water-Soil-Crop System in Seasonal Arid Region, Kunming University of Science and Technology, Kunming 650500, PR China

* Correspondence: kmyuanyong@163.com (Y. Y.), Tel.: +86-15887246439; 18292077095@163.com (J. G.), Tel.: +86-18292077095.

contents

Supplementary literature _A total of 71 articles were included in the Meta-analysis

Fig S.1 The frequency distribution of the logarithmic response ratio of soil organic carbon (SOC), total nitrogen (TN), total phosphorus (TP), available nitrogen (AN), available phosphorus (AP) content and Alkaline Phosphatase, Cellulase, Sucrase, Urease activity to biological crusts. The curve is a fitting curve obtained by Gaussian equation fitting.

Fig S.2 Funnel plot of logarithmic response ratio of soil organic carbon (SOC), total nitrogen (TN), total phosphorus (TP), available nitrogen (AN), available phosphorus (AP) content and Alkaline Phosphatase, Cellulase, Sucrase, Urease activity to biological crust, funnel plot effect value (natural logarithm of response ratio, ln R, horizontal axis) and standard error (SE, vertical axis).

Table S.1 The overall heterogeneity (Q_T) of the response of each explanatory variable to the biological crust, the percentage of heterogeneity caused by the real variation between the effect sizes (I^2), and the heterogeneity between groups (Q_B).

Table S.2 Kendall's tau rank correlation tests for assessing publication bias.

Table S.3 Rosenthal's fail-safe numbers for assessing publication bias.

Table S.4 The results of weighted regression analysis of the effect values (lnR) of response variables with annual average temperature, annual average rainfall and altitude.

Supplementary literature _A total of 71 articles were included in the Meta-analysis:

1. Zhao, H.-L.;Guo, Y.-R.;Zhou, R.-L.;Drake, S. Biological soil crust and surface soil properties in different vegetation types of Horqin Sand Land, China. *Catena.* **2010**, 82, 70-76.
2. Hu, R.;Wang, X.-p.;Pan, Y.-x.;Zhang, Y.-f.;Zhang, H. The response mechanisms of soil N mineralization under biological soil crusts to temperature and moisture in temperate desert regions. *European Journal of Soil Biology.* **2014**, 62, 66-73.
3. Guo, Y.;Zhao, H.;Zuo, X.;Drake, S.;Zhao, X. Biological soil crust development and its topsoil properties in the process of dune stabilization, Inner Mongolia, China. *Environmental Geology.* **2008**, 54, 653-662.
4. Xiao, B.;Veste, M. Moss-dominated biocrusts increase soil microbial abundance and community diversity and improve soil fertility in semi-arid climates on the Loess Plateau of China. *Applied Soil Ecology.* **2017**, 117, 165-177.
5. Liu, Y.;Yang, H.;Li, X.;Xing, Z. Effects of biological soil crusts on soil enzyme activities in revegetated areas of the Tengger Desert, China. *Applied Soil Ecology.* **2014**, 80, 6-14.
6. Gao, L.;Bowker, M.A.;Xu, M.;Sun, H.;Tuo, D.;Zhao, Y. Biological soil crusts decrease erodibility by modifying inherent soil properties on the Loess Plateau, China. *Soil Biology & Biochemistry.* **2017**, 105, 49-58.
7. Li, S.;Bowker, M.A.;Xiao, B. Biocrusts enhance non-rainfall water deposition and alter its distribution in dryland soils. *Journal of Hydrology.* **2021**, 595.
8. Gao, L.;Sun, H.;Xu, M.;Zhao, Y. Biocrusts resist runoff erosion through direct physical protection and indirect modification of soil properties. *Journal of Soils and Sediments.* **2020**, 20, 133-142.
9. Cheng, C.;Li, Y.;Long, M.;Gao, M.;Zhang, Y.;Lin, J.;Li, X. Moss biocrusts buffer the negative effects of karst rocky desertification on soil properties and soil microbial richness. *Plant and Soil.* **2022**, 475, 153-168.
10. Xu, H.;Zhang, Y.;Kang, B.;Qin, F.;Liu, X.;Zhou, H.;Shao, X. Different types of biocrusts affect plant communities by changing the microenvironment and surface soil nutrients in the Qinghai-Tibetan Plateau. *Arid Land Research and Management.* **2020**, 34, 306-318.
11. Liu, Y.;Cui, Z.;Huang, Z.;Miao, H.-T.;Wu, G.-L. The influence of litter crusts on soil properties and hydrological processes in a sandy ecosystem. *Hydrology and Earth System Sciences.* **2019**, 23, 2481-2490.
12. Liu, Y.;Xing, Z.;Yang, H. Effect of biological soil crusts on microbial activity in soils of the Tengger Desert (China). *Journal of Arid Environments.* **2017**, 144, 201-211.
13. Xie, T.;Shi, W.;Yang, H.;Lian, J.;Li, X. Variations in organic carbon mineralization of the biological soil crusts following revegetation in the Tengger Desert, North China. *Catena.* **2023**, 222.
14. Chen, Q.;Yan, N.;Xiong, K.;Zhao, J. Cyanobacterial diversity of biological soil crusts and soil properties in karst desertification area. *Frontiers in Microbiology.* **2023**, 14.
15. Yang, K.;Zhao, Y.;Gao, L. Biocrust succession improves soil aggregate stability of subsurface after "Grain for Green" Project in the Hilly Loess Plateau, China. *Soil & Tillage Research.* **2022**, 217.
16. Zhang, Y.;Gao, M.;Yu, C.;Zhang, H.;Yan, N.;Wu, Q.;Song, Y.;Li, X. Soil nutrients, enzyme activities, and microbial communities differ among biocrust types and soil layers in a degraded

karst ecosystem. *Catena.* **2022**, 212.

17. Zhang, E.;Hao, W.;Ma, Q. Effect of biological crust mulching on distribution characteristics and stability of soil aggregates in Loess Hilly Region. *Chinese Journal of Soil Science.* **2023**, 54, 606-613. (In Chinese)
18. Fan, J.;Wang, R.;Li, S.;Yu, H.;Huang, J. Enrichment law of atmospheric dustfall heavy metal pollution by different types of biocrusts in mining areas and its influencing factors. *Acta Ecologica Sinica.* **2023**, 43, 3168-3180. (In Chinese)
19. Liu, X.;Liu, J.;Chen, X.;Liang, H.;Liu, P.;Sharentuya. Community structure and diversity of biological soil crusts of Hobq Desert. *Journal of Inner Mongolia Forestry Science and Technology.* **2022**, 48, 21-27. (In Chinese)
20. Zhang, G.-H.;Yi, L.;Sun, B.-Y.;Li, J.-M.;Shen, S.-Y. Effects of moss biocrusts on soil-microbe-ectoenzyme stoichiometric characteristics in a subtropical area. *Chinese Journal of Applied Ecology.* **2022**, 33, 1791-1800. (In Chinese)
21. Pang, J.-W.;Bu, C.-F.;Guo, Q.;Ju, M.-C.;Jiang, M.;Mo, Q.-X.;Wang, H.-M. Spatial distribution and the influencing factors of organic carbon of biological crusts on regional scale in Mu Us sandy land, China. *Chinese Journal of Applied Ecology.* **2022**, 33, 1755-1763. (In Chinese)
22. Yao, H.;Wang, B.;An, S.;Yang, E.n.;Huang, Y. Variation in soil extracellular enzyme activities stoichiometry during biological soil crust formation in the Loess Plateau. *Arid Zone Research.* **2022**, 39, 456-468. (In Chinese)
23. Wang, F.;Xiao, B.;Li, S.;Sun, F. Biocrusts increase soil nutrient levels by increasing the nutrient retention ability of surface soil on the Loess Plateau. *Journal of Plant Nutrition and Fertilizers.* **2021**, 27, 1592-1602. (In Chinese)
24. Fan, J.;Li, S.;Du, Y.;Wang, R.;Yu, H.;Huang, J. Differences of bacterial communities in different biological soil crusts around thermal power plant and their influencing factors. *Chinese Journal of Applied Ecology.* **2021**, 32, 4107-4118. (In Chinese)
25. Zhang, Y.;Zhang, S.;Zhang, S.;Liu, Y.;Zhao, J.;Li, J. Effect of Moss Crust on Sandy Soil Properties and Bacterial Community in Mu Us Sandy Land. *Acta Pedologica Sinica.* **2021**, 58, 1585-1597. (In Chinese)
26. Zhang, S.;Wang, B.;Wang, X.;Huang, Q.;Yu, J.;Xu, M. Characteristics of nutrient and microbial nutrient limitation of different types of biological soil crusts on the vertical steep slopes of hilly region in the Loess Plateau. *Research of Soil and Water Conservation.* **2022**, 29, 51-57. (In Chinese)
27. Zheng, Z.;Xiong, K.;Rong, L.;Chi, Y. Effects of biological crusts on soil properties in karst rocky desertification areas of different levels. *Ecology and Environmental Sciences.* **2021**, 30, 1202-1212. (In Chinese)
28. Li, Y.;Bu, C.;Guo, Q.;Wei, Y. Ecological functions comparison of moss crust and algae crust in the Mu Us Sand Land. *Journal of Desert Research.* **2021**, 41, 138-144. (In Chinese)
29. Li, B.;Wu, Z.;Tao, Y.;Zhou, X.;Zhang, B. Effects of biological soil crust type on herbaceous diversity in the Gurbantunggut Desert. *Arid Zone Research.* **2021**, 38, 438-449. (In Chinese)
30. Sun, Y.;Feng, W.;Zhang, Y.;Qin, S.;Mao, H. Effects of biological soil crusts on soil enzyme activities of Artemisia ordosica community in the Mu Us Desert of northwestern China. *Journal of Beijing Forestry University.* **2020**, 42, 82-90. (In Chinese)
31. Zhou, H.;Wu, B.;Gao, Y.;Cheng, L.;Jia, X.;Pang, Y.;Zhao, H. Composition and influencing factors of the biological soil crust bacterial communities in the Sabina vulgaris community in

Mu Us Sandy Land. *Journal of Desert Research.* **2020**, 40, 130-141. (In Chinese)

32. Wang, F.-F.;Xiao, B.;Sun, F.-H.;Li, S.-L. Effects of biological soil crusts on solute transport characteristics of sandy and loessal soils on the Loess Plateau, China. *Chinese Journal of Applied Ecology.* **2020**, 31, 3404-3412. (In Chinese)

33. Li, Y.;Li, S.;Xiao, B.;Zhang, X.;Yao, X.;Wang, G. Study of soil water permeability and water flow characteristics under moss crusts covering the Loess Plateau. *Arid Zone Research.* **2020**, 37, 390-399. (In Chinese)

34. Wang, Y., Effects of moss-dominated biological soil crusts on soil enzyme activities and their correlations with soil physicochemical properties on the Loess Plateau of China, in, Shanxi University, 2018.

35. He, F.;Guo, C.;Wu, H.;Liu, Z.;Xu, W.;Jin, H. Effect of biological soil crust succession on soil texture, nutrient contents, and microbial populations of dune surfaces at the edge of the Minqin Oasis. *Acta Ecologica Sinica.* **2017**, 37, 6064-6073. (In Chinese)

36. Tao, Y.;Wu, G.;Liu, Y.;Zhang, Y. Soil stoichiometry and their influencing factors in typical shrub communities in the Gurbantunggut Desert,China. *Journal of Desert Research.* **2017**, 37, 305-314. (In Chinese)

37. Xu, B.-x.;Chen, Y.-l.;Hu, Y.-g.;Zhang, Z.-s.;Li, G.;Li, M.-r.;Chen, D. Nitrification of biological soil crusts and soil system during drought process and its response to temperature and moisture: A case study in the Shapotou region, Northwest China. *Chinese Journal of Applied Ecology.* **2015**, 26, 1113-1120. (In Chinese)

38. Yang, H.;Liu, Y.;Wang, T. Effects of biological soil crusts on soil enzyme activities in desert area. *Acta Pedologica Sinica.* **2015**, 52, 654-664. (In Chinese)

39. Song, Y.;Gong, Y.;Liao, B.;Liu, W. Nitrogen fixation of biological soil crusts on copper mining tailings and its influence factors. *Acta Pedologica Sinica.* **2011**, 48, 701-707. (In Chinese)

40. Meng, J.;Bu, C.;Li, L.;Zhang, X.;Sun, Z.;Zhang, X. Effects of biological soil crust on slope soil carbon and nitrogen under erosion conditions. *Science of Soil and Water Conservation.* **2011**, 9, 45-51. (In Chinese)

41. Meng, J.;Bu, C.;Zhao, Y.;Zhang, X. Effects of biological soil crust on soil enzyme activities and nutrients content in wind-water erosion crisscross region, Northern Shaanxi Province, China. *Journal of Natural Resources.* **2010**, 25, 1964-1974. (In Chinese)

42. Chen, Z.;Yang, G.-d.;Sun, Q.-y. Effects of bio-crust on soil microbial biomass and enzyme activities in copper mine tailings. *Chinese Journal of Applied Ecology.* **2009**, 20, 2193-2198. (In Chinese)

43. Zhao, H.-L.;Guo, Y.-R.;Zhou, R.-L.;Zhao, X.-Y. Effects of vegetation cover on physical and chemical properties of bio-crust and under-layer soil in Horqin Sand Land. *Chinese Journal of Applied Ecology.* **2009**, 20, 1657-1663. (In Chinese)

44. Bo, X.;YunGe, Z.;Ming'An, S. Effects of biological soil crust on soil physicochemical properties in water-wind erosion crisscross region, northern Shaanxi Province, China. *Acta Ecologica Sinica.* **2007**, 27, 4662-4670. (In Chinese)

45. YuanMing, Z.;WeiKang, Y.;XueQin, W.;DaoYuan, Z. Influence of cryptogamic soil crusts on accumulation of soil organic matter in Gurbantunggut Desert, northern Xinjiang, China. *Acta Ecologica Sinica.* **2005**, 25, 3420-3425. (In Chinese)

46. Gao, L.;Zhao, Y.;Xu, M.;Sun, H.;Yang, Q. The effects of biological soil crust succession on soil ecological stoichiometry characteristics. *Acta Ecologica Sinica.* **2018**, 38, 678-688. (In Chinese)

47. Zhou, W., Effect of biocrusts on Heavy metal enrichment and bioavailability in Qinling Mining Are, in, Northwest A&F Univers, 2023.
48. Guo, Q., Study on organic carbon characteristics and carbon conversion process of Biological crusts in Mu Us Sandland, in, Northwest A＆F University, 2022.
49. Liang, L., Bacterial diversity of biological soil crust and related ecological function in Mu Us Sandy Land, in, Chinese Academy of Forestry, 2021.
50. Li, B., The study of characteristics of photosynthetic and soil CO2 flux of biocrusts in MU US sandland, in, Northwest A＆F University, 2019.
51. Li, Y., Study on Characteristics of Soil Nutrients and Carbon Storage in Biocrusts and Underlying Soil of Mu Us Sandland, in, Northwest A＆F University, 2018.
52. Sun, P., Effect of biological soil crust on ecological restoration of coal mine dump in grassland in, China University of Geosciences (Beijing), 2018.
53. Chen, L., The characteristics of moss crusts of Loess Plateau of China and moss crusts their on soil physicochemical properties and water repellency, in, Shenyang Agriculture University, 2017.
54. Han, B., The study of biological soil crusts development and its microhabiats soil nutrients in steppe grasslands of the Loess Plateau, China, in, Lanzhou University, 2021.
55. Zhao, Y., Population characteristics of biological crusts and its effects on topsoil biological activities in Loss Area of Northern Shaanxi, in, Beijing Forestry University, 2014.
56. Ren, H., Influence of biological soil crusts on spatial and temporal heterogeneity of soil fertility in Kubuqi Desert, Inner Mongolia, China, in, Inner Mongolia Normal University, 2011.
57. Ping, Y.;Tan, T.;Li, Y.;Zeng, J.;Mi, G.;Tan, L.;Guo, Z. Soil properties change and soil detachment response driven by biocrusts in typical small watershed of Danjiangkou Reservoir Area. *Journal of Soil and Water Conservation.* **2023**, 37, 87-94. (In Chinese)
58. Cao, Y.;Xiao, B.;Jiang, Z.;Li, S.;Sun, F. Effects of biocrusts development on base cations release and mineral weathering in aeolian sandy soil. *Journal of Soil and Water Conservation.* **2022**, 36, 77-85. (In Chinese)
59. Qin, F.;Kang, B.;Jiang, F.;Xu, H.;Zhou, H.;Wei, X.;Liu, X.;Shao, X. Effects of biological crusts succession on soil microbial communities in alpine steppe. *Acta Agrestia Sinica.* **2019**, 27, 832-840. (In Chinese)
60. Li, S.;Xiao, B.;Sun, F. Characteristics of water vapor sorption and condensation in biocrusts covered surface soil in arid and semiarid areas of the Loess Plateau, China. *Transactions of the Chinese Society of Agricultural Engineering.* **2020**, 36, 111-119. (In Chinese)
61. Wu, Y.;Rao, B.;Wu, P.;Liu, Y.;Li, G.;Li, D. Development of artificially induced biological soil crusts in fields and their effects on top soil. *Plant and Soil.* **2013**, 370, 115-124.
62. Jia, C.;Liu, Y.;He, H.;Miao, H.-t.;Huang, Z.;Zheng, J.;Han, F.;Wu, G.-L. Formation of litter crusts and its multifunctional ecological effects in a desert ecosystem. *Ecosphere.* **2018**, 9.
63. Yao, C., The fate of biological soil crusts fixed nitrogen in the Loess Plateau Region, China, in, Northwest A＆F University, 2015.
64. Xie, T.;Li, Y.;Li, X. Organic carbon mineralization of biological soil crusts and subsoils in the revegetated areas of the southeast fringe of the Tengger Desert. *Acta Ecologica Sinica.* **2021**, 41, 2339-2348. (In Chinese)
65. Sun, H.;Li, X.;Jin, L.;Li, C.;Zhang, J. Effects of biological soil crusts on the physical and chemical properties of soil and vegetation of artificial grassland in the Yellow River Source

Zone. *Acta Agrestia Sinica.* **2020**, 28, 509-520. (In Chinese)
66. Zhang, J.;Wu, B. Influences of biological soil crusts on physicochemical properties of soil in artemisia ordosica and sabina vulgaris communities. *Journal of North-East Forestry University.* **2012**, 40, 58-61. (In Chinese)
67. Hu, Y.;Hou, Y.;Li, Y. Effects of biological crusts on soil erosion and nutrient in red soil. *Guizhou Agricultural Sciences.* **2015**, 43, 114-119. (In Chinese)
68. Sun, F.;Xiao, B.;Zhang, X.;Wang, G.;Li, S.;Yao, X. Effects of biocrust covering on soil water infiltration characteristics on the Loess Plateau and its simulation. *Journal of Northwest A & F University. Natural Science Edition.* **2020**, 48, 82-91. (In Chinese)
69. Zhang, S.;Gao, H.;Yan, D.;Huang, H. Effects of desert biological soil crusts succession on microbial community structure and soil enzyme activities. *Journal of Desert Research.* **2023**, 43, 178-187. (In Chinese)
70. Li, Y.;Liu, M.;Sun, Q. Comparing the fungal community structure in biological soil crusts obtained under different plant communities in a copper mine tailings. *Acta Ecologica Sinica.* **2016**, 36, 5884-5892. (In Chinese)
71. Wu, Z., Relationship between soil nutrients of carbon and nitrogen and microbial activity in different successional stages of biological crusts in typical hilly areas of the Loess Platea, in, Shanxi Normal University, 2022.

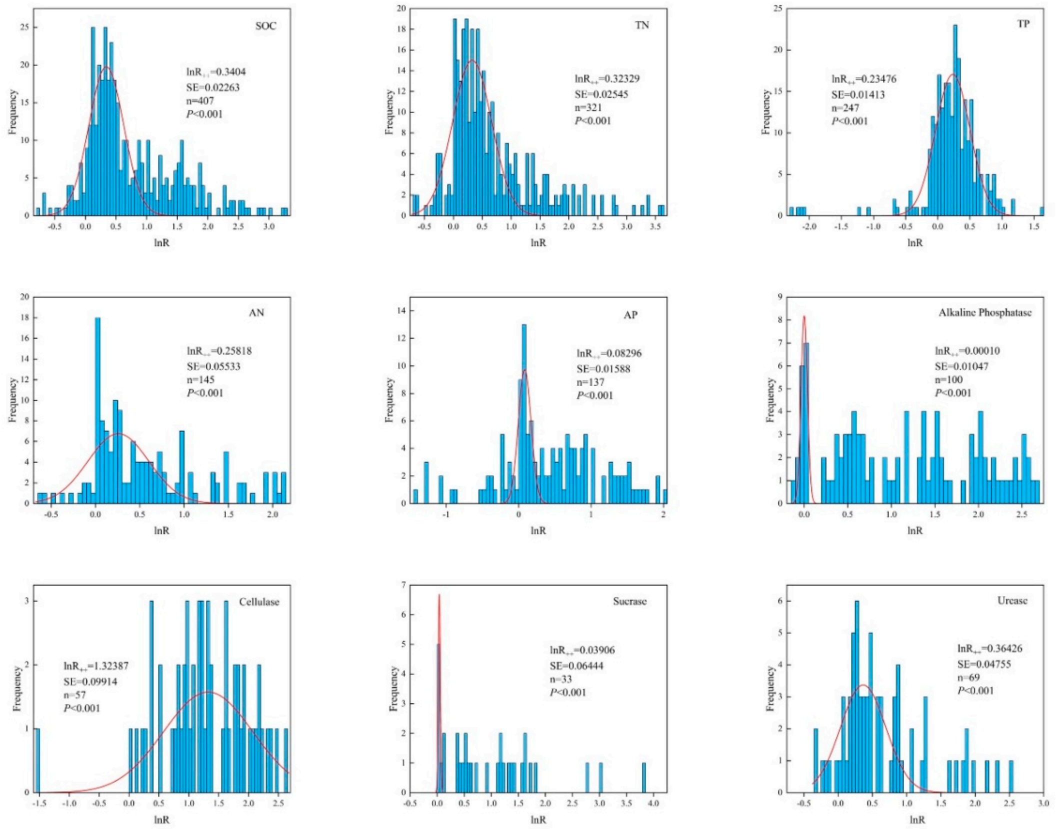

Fig. S.1 The frequency distribution of the logarithmic response ratio of soil organic carbon (SOC), total nitrogen (TN), total phosphorus (TP), available nitrogen (AN), available phosphorus (AP) content and Alkaline Phosphatase, Cellulase, Sucrase, Urease activity to biological crusts. The curve is a fitting curve obtained by Gaussian equation fitting.

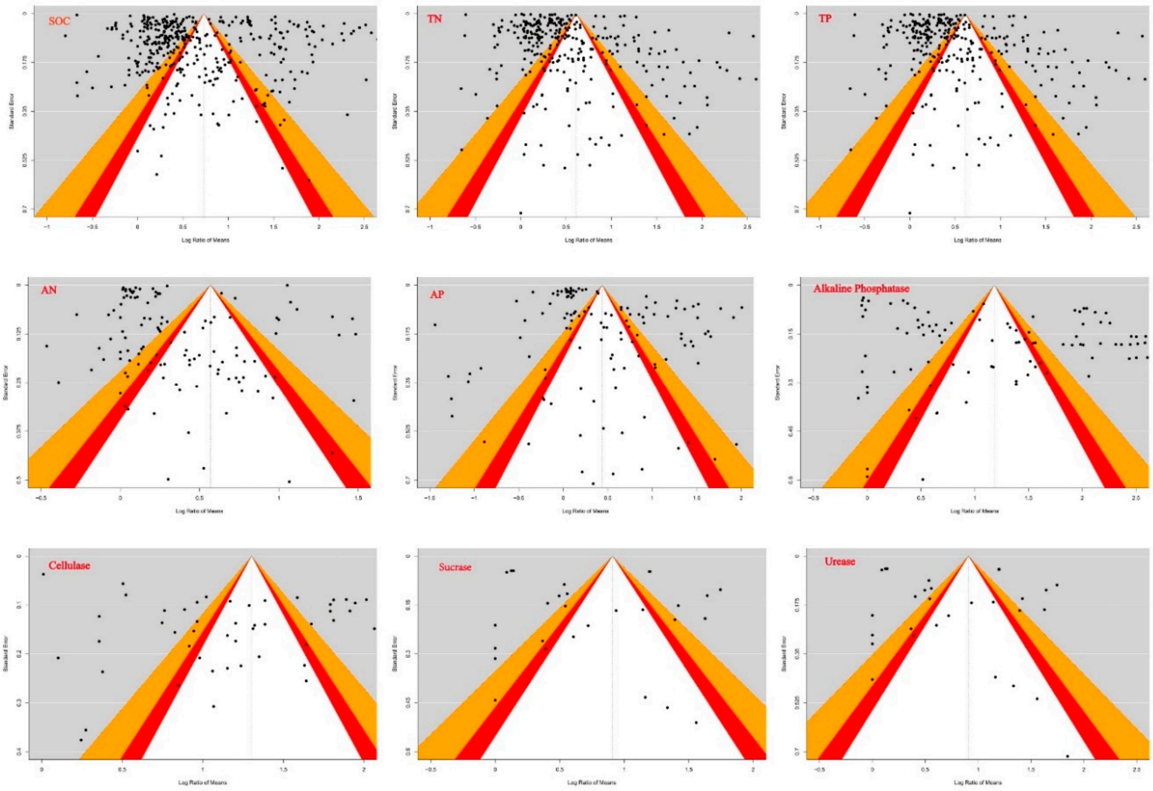

Fig. S.2 Funnel plot of logarithmic response ratio of soil organic carbon (SOC), total nitrogen (TN), total phosphorus (TP), available nitrogen (AN), available phosphorus (AP) content and Alkaline Phosphatase, Cellulase, Sucrase, Urease activity to biological crust, funnel plot effect value (natural logarithm of response ratio, ln R, horizontal axis) and standard error (SE, vertical axis). There is a 90 % confidence interval within white, a 95 % confidence interval within red, and a 99 % confidence interval within orange.

Table S.1 The overall heterogeneity (Q_T) of the response of each explanatory variable to the biological crust, the percentage of heterogeneity caused by the real variation between the effect sizes (I^2), and the heterogeneity between groups (Q_B).

Response variable	N	Q_T	I^2	Q_B						
				soil texture	crust type	Ecosystem type	soil depth	annual average rainfall	annual average temperature	altitude
SOC	407	165298.71***	99.75	31.07***	16.92**	68.06***	17.28**	94.65***	40.49***	19.49**
TN	321	185214.53***	99.83	37.67***	30.93***	90.64***	43.51***	95.20***	61.22***	31.17***
TP	247	79578.03***	99.69	29.16***	15.85***	8.73	0.76	13.71*	9.04*	9.89**
AN	145	10648.01***	98.65	20.47***	57.46***	50.34***	19.03**	71.31***	39.68***	70.02***
AP	137	6198.03***	97.81	77.87***	94.12***	13.23**	31.46***	279.58***	83.77***	1.11**
Alkaline Phosphatase	100	5448.40***	98.18	NA	7.26*	49.88***	81.72***	76.19***	6.87**	NA
Cellulase	57	2423.11***	97.69	NA	7.28***	NA	NA	NA	2.24**	NA
Sucrase	33	1216.52***	97.37	NA	NA	NA	NA	NA	NA	NA
Urease	69	4854.63***	98.60	NA	1.96	3.02	7.91	7.23*	NA	NA

* $P < 0.05$; ** $P < 0.01$; *** $P < 0.001$.

NA: Only one group of studies is greater than or equal to 10, not grouped

Table S.2 Kendall's tau rank correlation tests for assessing publication bias.

	Response variable	OC	TN	TP	AN	AP	Alkaline Phosphatase	Cellulase	Sucrase	Urease
Overall		0.7190	0.1447	0.3840	0.0001	0.8971	0.5773	0.1250	0.8769	0.3940
soil texture	coarse	0.0953	0.0001	0.2968	ND	ND	NA	NA	NA	NA
	medium	0.0002	0.6297	0.0001	0.0001	0.6118	NA	NA	NA	NA
	fine	0.0768	0.9202	0.1424	0.6115	0.7080	NA	NA	NA	NA
crust type	algae	0.2961	0.5832	0.9180	0.0092	0.7665	ND	ND	NA	NA
	mix	0.0002	0.4354	0.1125	0.8376	0.8955	0.8348	0.2176	NA	NA
	lichen	0.6966	0.1491	0.0090	0.0239	0.7428	ND	ND	NA	NA
	moss	0.0002	0.0022	0.4627	0.3017	0.1619	0.0093	0.9013	NA	NA
ecosystem	desert	0.0001	0.0010	NA	0.7130	0.6911	0.0026	NA	NA	NA
	grassland	0.0001	0.0534	NA	0.2317	0.3502	0.5077	NA	NA	NA
	forest	0.8205	0.2091	NA	ND	0.4816	ND	NA	NA	NA
soil depth	0 - 5cm	0.0287	0.0099	NA	0.0020	0.5896	0.4263	NA	NA	NA
	5 - 10cm	0.0101	0.2206	NA	0.6809	0.2269	ND	NA	NA	NA
	>10cm	0.3601	0.7708	NA	ND	0.5453	0.0390	NA	NA	NA
annual average rainfall	0 - 250mm	0.9140	0.6436	0.3958	ND	0.1375	0.0060	NA	NA	0.8031
	250 - 400mm	0.0001	0.0007	0.0087	0.5243	0.5980	ND	NA	NA	ND
	400 - 800mm	0.0001	0.8662	0.0001	0.0660	0.8208	0.6558	NA	NA	0.7160
	>800mm	0.1875	0.3620	0.0001	0.9546	0.9803	ND	NA	NA	ND
annual average temperature	≤10℃	0.0223	0.0318	0.0002	0.0131	0.6599	0.9670	0.3881	NA	NA
	>10℃	0.8627	0.2075	0.0001	0.9590	0.9806	0.1438	0.2106	NA	NA
altitude	≤500m	0.3597	0.0646	0.0035	0.9502	0.2318	NA	NA	NA	NA
	>500m	0.7903	0.0076	0.3458	0.0001	0.9862	NA	NA	NA	NA

ND: Effective grouping, but the number of studies within the group at this level is less than 10.

NA: Only one group of studies is greater than or equal to 10, not grouped.

Table S.3 Rosenthal's fail-safe numbers for assessing publication bias.

	Response variable	OC	TN	TP	AN	AP	Alkaline Phosphatase	Cellulase	Sucrase	Urease
Overall		47783.30	51098.21	2243.6	4626.60	6574.73	4254.29	2203.88	460.36	809.82
soil texture	coarse	2047.75	586.27	321.21	ND	ND	NA	NA	NA	NA
	medium	24915.07	38052.37	11831.33	4375.47	4943.02	NA	NA	NA	NA
	fine	NS	NS	NS	NS	NS	NA	NA	NA	NA
crust type	algae	4036.26	2509.11	NS	NS	NS	ND	NS	NA	NA
	mix	1972.87	3370.67	NS	169.20	167.31	907.20	347.04	NA	NA
	lichen	480.66	862.83	NS	390.00	NS	ND	ND	NA	NA
	moss	5019.00	7314.09	842.86	1224.42	3014.48	836.58	991.06	NA	NA
ecosystem	desert	13538.29	14206.53	NA	2135.65	625.15	2095.17	NA	NA	NA
	grassland	10545.14	12575.99	NA	3920.27	1621.76	509.80	NA	NA	NA
	forest	22.62*	0.0782*	NA	ND	14.11*	ND	NA	NA	NA
soil depth	0 - 5cm	19405.70	26093.59	NA	3345.40	3349.81	340.15	NA	NA	NA
	5 - 10cm	1875.64	744.24	NA	548.13	216.17	ND	NA	NA	NA
	>10cm	259.40	120.74	NA	ND	NS	2431.65	NA	NA	NA
annual average rainfall	0 - 250mm	385.20	2351.19	91.31	ND	143.63	2683.12	NA	NA	159.69
	250 - 400mm	8413.00	7691.23	2494.17	2000.46	277.69	ND	NA	NA	ND
	400 - 800mm	9721.46	8382.68	2495.72	618.62	671.44	400.70	NA	NA	230.79
	>800mm	484.24	590.09	NS	NS	NS	ND	NA	NA	ND
annual average temperature	≤10℃	37725.63	41516.15	6930.53	5239.64	2078.21	1965.28	565.67	NA	NA
	>10℃	1421.45	1110.65	NS	NS	NS	543.11	1015.00	NA	NA
altitude	≤500m	1578.47	3509.84	2982.36	1579.25	154.36*	NA	NA	NA	NA
	>500m	33325.70	26688.10	486.59*	1128.60	4199.17	NA	NA	NA	NA

NS: The effect of biological crust on the response variable is not significant, and there is no fail-safe number.

ND: Effective grouping, but the number of studies within the group at this level is less than 10.
NA: Only one group of studies is greater than or equal to 10, not grouped.

Table S.4 The results of weighted regression analysis of the effect values(lnR) of response variables with annual average temperature, annual average rainfall and altitude.

Response variable	N	annual average temperature			annual average rainfall			altitude		
		intercept	slope	P	intercept	slope	P	intercept	slope	P
SOC	407	1.2869	-0.0612	0.0001	1.2783	-0.0011	0.0001	0.9308	-0.0002	0.0035
TN	321	1.0315	-0.0452	0.0072	1.0527	-0.0009	0.0001	0.9348	-0.0003	0.0080
TP	245	0.3132	-0.0102	0.1010	0.3427	-0.0002	0.0115	0.3990	-0.0002	0.0001
AN	145	1.1158	-0.0586	0.0001	1.0827	-0.0010	0.0001	0.9976	-0.0004	0.0001
AP	137	0.6918	-0.0267	0.0676	0.8280	-0.0008	0.0004	0.3482	0.0001	0.6362
Alkaline Phosphatase	100	0.9349	0.0245	0.8231	1.6392	-0.0012	0.0001	0.4773	0.0006	0.3852
Cellulase	57	-2.0024	0.3341	0.6779	2.4641	-0.0056	0.0117	-4.5458	0.0046	0.9492
Sucrase	33	1.5709	-0.0754	0.5835	1.5462	-0.0014	0.3771	1.1418	-0.0002	0.9846
Urease	69	0.2897	0.0386	0.2815	0.7582	-0.0002	0.3012	0.4739	0.0002	0.5678
